# Preventive effect of prednisolone on permanent hypothyroidism in subacute thyroiditis: retrospective study of 743 cases

**DOI:** 10.1210/jendso/bvag187

**Published:** 2026-08-12

**Authors:** Ai Suzuki, Natsuko Watanabe, Jaeduk Yoshimura Noh, Hideyuki Imai, Shigenori Hiruma, Aya Kinoshita, Azusa Aida, Nami Suzuki, Miho Fukushita, Masako Matsumoto, Ai Yoshihara, Kiminori Sugino, Koichi Ito

**Affiliations:** Department of Internal Medicine, Ito Hospital, 4-3-6 Jingumae, Shibuya-ku, Tokyo 150-8308, Japan; Department of Internal Medicine, Ito Hospital, 4-3-6 Jingumae, Shibuya-ku, Tokyo 150-8308, Japan; Department of Internal Medicine, Ito Hospital, 4-3-6 Jingumae, Shibuya-ku, Tokyo 150-8308, Japan; Department of Internal Medicine, Ito Hospital, 4-3-6 Jingumae, Shibuya-ku, Tokyo 150-8308, Japan; Department of Internal Medicine, Ito Hospital, 4-3-6 Jingumae, Shibuya-ku, Tokyo 150-8308, Japan; Department of Internal Medicine, Ito Hospital, 4-3-6 Jingumae, Shibuya-ku, Tokyo 150-8308, Japan; Department of Internal Medicine, Ito Hospital, 4-3-6 Jingumae, Shibuya-ku, Tokyo 150-8308, Japan; Department of Internal Medicine, Ito Hospital, 4-3-6 Jingumae, Shibuya-ku, Tokyo 150-8308, Japan; Department of Internal Medicine, Ito Hospital, 4-3-6 Jingumae, Shibuya-ku, Tokyo 150-8308, Japan; Department of Internal Medicine, Ito Hospital, 4-3-6 Jingumae, Shibuya-ku, Tokyo 150-8308, Japan; Department of Internal Medicine, Ito Hospital, 4-3-6 Jingumae, Shibuya-ku, Tokyo 150-8308, Japan; Department of Surgery, Ito Hospital, 4-3-6 Jingumae, Shibuya-ku, Tokyo 150-8308, Japan; Department of Surgery, Ito Hospital, 4-3-6 Jingumae, Shibuya-ku, Tokyo 150-8308, Japan

**Keywords:** glucocorticoid, nonsteroidal anti-inflammatory drugs, permanent hypothyroidism, prednisolone, subacute thyroiditis

## Abstract

**Purpose:**

Permanent hypothyroidism (PH) after subacute thyroiditis (SAT) requires lifelong thyroid hormone replacement therapy. This study aimed to investigate the risk factors for PH and its association with treatment.

**Methods:**

A retrospective observational study of 743 patients diagnosed with SAT from 2016 to 2019 was conducted.

**Results:**

Initial treatment was prednisolone (PSL) in 332 patients (44.7%), nonsteroidal anti-inflammatory drugs (NSAIDs) in 364 (49.0%), and rest without medication in 47 (6.3%). Of the NSAID-treated patients, 238 (65.4%) switched to PSL. Final treatment was PSL in 570 (76.7%), NSAIDs in 126 (17.0%), and rest in 47 (6.3%). Permanent hypothyroidism occurred in 32 patients (4.3%). Prednisolone therapy was used less frequently in the PH group (46.9%) than in the euthyroid group (78.1%, *P* < .01). Multivariable logistic regression identified no PSL therapy (odds ratio [OR] 3.92, 95% confidence interval [CI] 1.88-8.24, *P* < .01) and male sex (OR 2.52, 95% CI 1.13-5.37, *P* = .03) as significant risk factors for PH.

Compared with patients aged <70 years, those ≥70 years had significantly lower free triiodothyronine, free thyroxine, and C-reactive protein levels, but higher thyroid-stimulating hormone levels. The proportion of elderly patients receiving PSL therapy was lower (56.1% vs 77.9%; *P* < .01), and the duration of PSL therapy was shorter (*P* = .01).

**Conclusion:**

Prednisolone therapy for SAT helps prevent PH through its anti-inflammatory effects. In younger patients, early PSL therapy may be preferable to reduce the risk of PH, as PH requires long-term levothyroxine therapy. In elderly patients, NSAIDs were often sufficient, whereas PSL therapy may be considered when clinically indicated.

Subacute thyroiditis (SAT) is an inflammatory disease of the thyroid gland. Its incidence is about 2.4 to 4.9 cases per 100 000 population/year (1, 2), and the female-to-male ratio is approximately 4:1 (3, 4). Patients typically experience painful swelling of the thyroid gland. The pain often begins on one side of the neck and may then migrate to the other side. Fever may also occur. On thyroid ultrasound, a hypoechoic area is seen in the painful region. Blood tests typically show increased C-reactive protein (CRP) and erythrocyte sedimentation rate (ESR). Subacute thyroiditis leads to thyrotoxicosis because of destructive thyroiditis. This is usually followed by a hypothyroid phase, and normal thyroid function eventually returns in most patients. However, some patients develop permanent hypothyroidism (PH), the underlying mechanism of which is not fully understood. Several factors, such as age, sex, thyroid function, inflammatory markers, and antithyroid antibodies, have been studied, but their associations with PH remain inconsistent (4-6). The extent of the hypoechoic area on thyroid ultrasound may indicate the risk of developing hypothyroidism after SAT (7). However, another study suggested that the hypoechoic area reflects the severity of inflammation and thyroid hormone levels, but does not predict PH (8).

Subacute thyroiditis is commonly treated with nonsteroidal anti-inflammatory drugs (NSAIDs) in mild cases and oral glucocorticoids in severe cases. However, there are no clear guidelines on how to choose between NSAIDs and glucocorticoids. To date, few studies have examined the relationships between these treatments and PH, and no consensus has been reached. Since PH after recovery from SAT requires lifelong thyroid hormone replacement therapy, its causes and prevention need to be clarified. Therefore, this study examined the clinical characteristics of patients who developed PH after SAT and their association with treatment at our hospital.

## Materials and methods

### Study design and patient selection

This study included 1380 patients who were diagnosed with SAT at their first visit to our hospital between January 2016 and December 2019. The study was approved by Ito Hospital (approval number 329).

### Diagnosis

According to the diagnostic guidelines for SAT published by the Japan Thyroid Association (9), the diagnosis is confirmed when all of the following are present: a painful goiter as a clinical finding; elevated CRP or ESR levels on laboratory testing; signs of thyrotoxicosis, such as high free thyroxine (FT4) and low thyroid-stimulating hormone (TSH) levels; and hypoechoic areas corresponding to the painful region on thyroid ultrasound. If only a painful goiter, elevated CRP or ESR levels, and signs of thyrotoxicosis are present, the condition is classified as suspected SAT.

However, in clinical practice, there are cases in which a painful goiter is present and thyroid ultrasound findings are consistent with SAT, but no thyrotoxicosis is evident at the first visit. In this study, such cases were also included when the clinical findings were still considered consistent with SAT. In addition, cases with a painful goiter and ultrasound findings typical of SAT, but without CRP measurements at the first visit, were included if later CRP values or the clinical course did not contradict the diagnosis of SAT.

Cases with antithyroglobulin antibody (TgAb) ≥ 4000 IU/mL or antithyroid peroxidase antibody (TPOAb) ≥ 600 IU/mL, which exceeded our measurement limit, were also excluded to help differentiate these cases from painful Hashimoto thyroiditis (pHT). Of the patients who were positive for TgAb or TPOAb at the first visit, those who met at least one of the following criteria were included: negative conversion of antibodies, reduction of thyroid volume to <20 mL on ultrasound or disappearance of the goiter on palpation after recovery from SAT, or recovery within 90 days.

### Treatment

Treatment was chosen by the attending physicians, who selected prednisolone (PSL), NSAIDs, or rest without medication. Patients who started with NSAIDs were switched to PSL if their symptoms worsened, or if the effect was insufficient, based on the physician's judgment. The initial dose was selected by the attending physician, and the dosage was adjusted according to the patient's symptoms, and then gradually reduced and stopped.

### Definition of permanent hypothyroidism

Permanent hypothyroidism was defined as overt hypothyroidism or subclinical hypothyroidism with TSH ≥ 10 mIU/L persisting for ≥9 months after completion of SAT treatment, or the requirement for ongoing levothyroxine (LT4) replacement therapy. The need for ongoing LT4 therapy was defined by an increase in TSH levels despite LT4 administration, or the development of hypothyroidism when LT4 was reduced or discontinued. The rationale for defining PH as persisting for ≥9 months after completion of SAT treatment is explained in “Thyroid function after recovery from subacute thyroiditis.” Patients with PH were defined as the PH group. Patients with euthyroidism or subclinical hypothyroidism with TSH < 10 mIU/L were defined as the E group.

### Laboratory measurements

Free triiodothyronine (FT3) and FT4 levels were measured by electrochemiluminescence immunoassays (ECLIAs) (Elecsys FT3, RRID:AB_3741142 and RRID:AB_3752389, and Elecsys FT4, RRID:AB_3095310 and RRID:AB_3752390; Roche Diagnostics GmbH, Basel, Switzerland; reference ranges at our hospital: 2.2-4.3 pg/mL and 0.8-1.6 ng/dL, respectively). Thyroid-stimulating hormone was measured using an ECLIA (Elecsys TSH, RRID:AB_3095311 and RRID:AB_3752391; Roche Diagnostics GmbH, Basel, Switzerland; reference range at our hospital: 0.2-4.5 mIU/L). Antithyroglobulin antibody and TPOAb levels were determined using an ECLIA (Elecsys Anti-Tg, RRID:AB_3752392 and RRID:AB_3752393, and Elecsys Anti-TPO, RRID:AB_2916057 and RRID:AB_3675830; Roche Diagnostics; reference values at our hospital: TgAb cutoff ≤ 40 IU/mL and TPOAb cutoff ≤ 28 IU/mL).

Thyroid volume was estimated on ultrasonography by measuring the length, width, and depth of each lobe of the thyroid gland in millimeters and calculating the volume according to the following formula: thyroid volume = ([0.7365 × right lobe length × width × depth] + [0.7412 × left lobe length × width × depth]) − 0.55 (10).

### Statistical analysis

For statistical analyses, patients were classified into different subgroups according to thyroid function outcome after SAT (E and PH groups), PSL therapy status (with and without PSL therapy), sex, and age (<70 years and ≥70 years), as appropriate for each analysis.

Categorical variables are expressed as numbers (percentages), and were compared using Pearson's χ² test or Fisher's exact test, as appropriate. Continuous variables in the present analysis did not follow a normal distribution and are expressed as medians (ranges); comparisons were made using the Wilcoxon rank-sum test. Statistical significance was set at *P* < .05. To evaluate factors associated with PH, multivariable logistic regression analysis was performed using sex, age, and PSL therapy as explanatory variables. Sensitivity analyses were conducted by adding FT4 alone or both FT4 and CRP to the main multivariable model. An additional sensitivity analysis restricted to patients aged <70 years was also performed.

All statistical analyses were conducted using JMP 12 (SAS Institute Inc., Cary, NC, USA).

## Results

### Clinical characteristics

**Figure 1:**
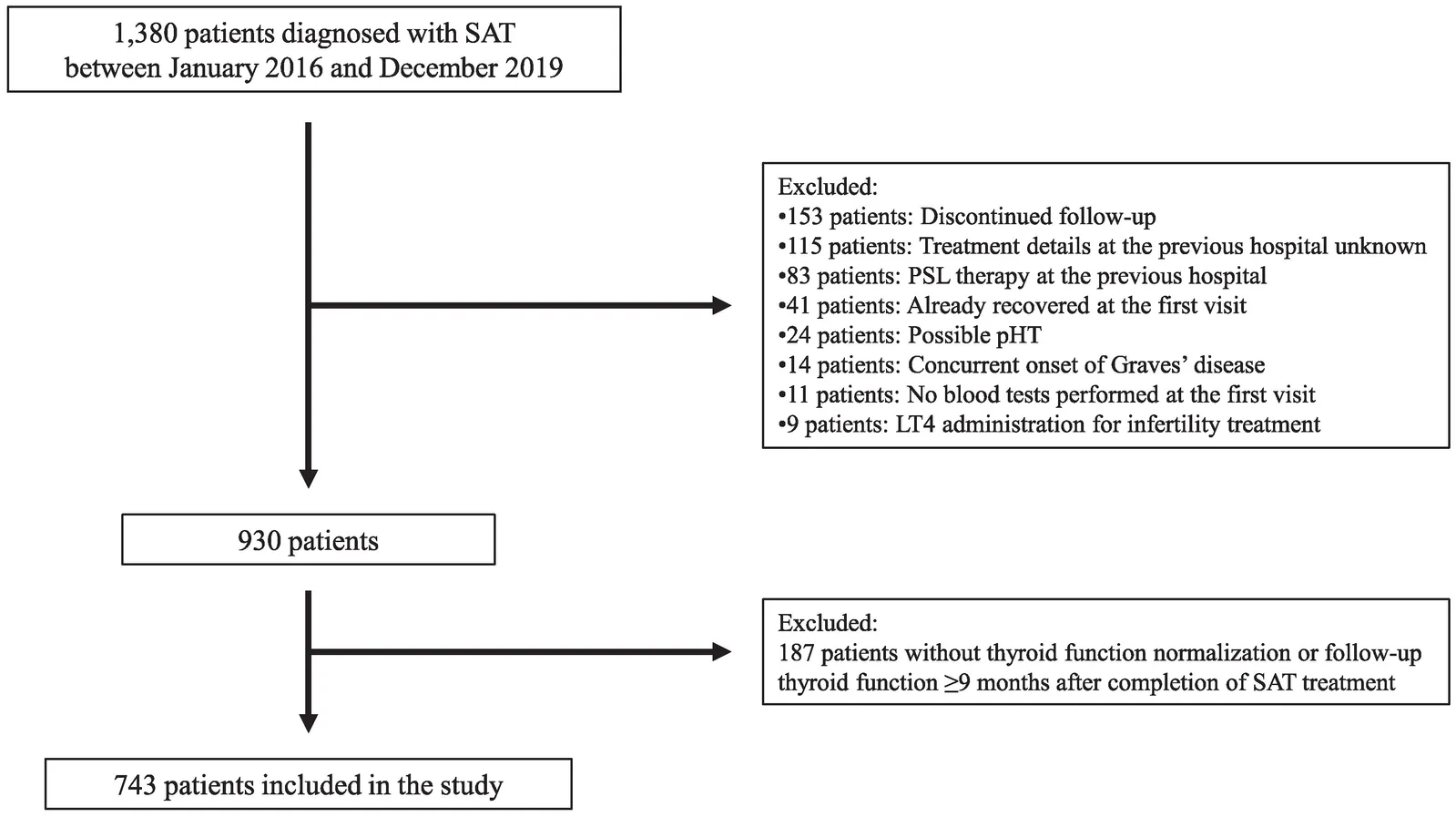
Patient selection flowchart. LT4, levothyroxine; pHT, painful Hashimoto thyroiditis; PSL, prednisolone; SAT, subacute thyroiditis.

Of the 1380 patients diagnosed with SAT, 450 were excluded for the following reasons: discontinued follow-up (n = 153), unknown treatment details at the previous hospital (n = 115), PSL therapy at the previous hospital (n = 83), already recovered at the first visit (n = 41), possible pHT (n = 24), concurrent onset of Graves disease (n = 14), no blood tests performed at the first visit (n = 11), and LT4 administration for infertility treatment (n = 9). Of the remaining 930 patients, 743 with normalized thyroid function or thyroid function confirmed at ≥9 months after completion of SAT treatment were included (Fig. 1).

The baseline characteristics of the 743 patients are shown in Table 1. Females accounted for 82.2% of the study population. Some laboratory measurements and thyroid volume data were not available for all patients.

**Table 1:** Baseline characteristics of patients with SAT and comparison between the E and PH groups

	Total	E group	PH group	*P* value
N	743	711	32	
Female/male, n	611/132	590/121	21/11	.01
Age (y)	48 (24-83)	48 (24-83)	47 (33-81)	.54
FT3 (pg/mL)	6.6 (2.4-28.2)	6.5 (2.5-28.2)	7.3 (2.4-24.0)	.20
FT4 (ng/dL)	2.6 (0.9-7.8)	2.6 (0.9-7.8)	2.9 (1.2-7.8)	.31
TSH (mIU/L)	0.01 (0.01-3.93)	0.01 (0.01-3.93)	0.01 (0.01-1.15)	.05
CRP (mg/dL)	n = 641	n = 612	n = 29	
	3.3 (0.2-25.1)	3.3 (0.2-25.1)	2.4 (0.2-16.0)	.23
TgAb (IU/mL)	n = 733	n = 702	n = 31	
	15.0 (10-2366)	15.1 (10-2366)	13.4 (10-504.2)	.26
TgAb positivity, n (%)	161/733 (22.0%)	155/702 (22.1%)	6/31 (19.4%)	.83
TPOAb (IU/mL)	n = 733	n = 702	n = 31	
	10.4 (5-528)	10.4 (5-528)	11.0 (5.6-290)	.54
TPOAb positivity, n (%)	45/733 (6.1%)	43/702 (6.1%)	2/31 (6.5%)	1.00
Thyroid volume (mL)	n = 421	n = 402	n = 19	
	24.5 (5.1-70.8)	24.5 (9.1-70.8)	22.9 (5.1-37.9)	.61

Data are presented as median (range).

Abbreviations: CRP, C-reactive protein; E group, euthyroid group; FT3, free triiodothyronine; FT4, free thyroxine; PH group, permanent hypothyroidism group; SAT, subacute thyroiditis; TgAb, antithyroglobulin antibody; TPOAb, antithyroid peroxidase antibody; TSH, thyroid-stimulating hormone.

### Sex differences

Comparisons by sex are shown in Table 2. The median age of SAT patients was significantly higher in male than in female patients, 50 (27-83) vs 48 (24-83) years (*P* = .02). Male patients also had significantly higher FT4 levels (*P* = .03), lower TSH levels (*P* < .01), and higher CRP levels (*P* < .01). Free triiodothyronine levels tended to be higher in male patients (*P* = .06). There were no significant differences in TgAb or TPOAb levels or positivity rates between the sexes, whereas thyroid volume was significantly greater in male patients (*P* < .01). There was no difference in the rate of PSL therapy by sex, but male patients had a longer interval from symptom onset to initiation of PSL therapy (*P* < .05).

**Table 2:** Comparison of clinical characteristics by sex

	Male		Female		
	N		n		*P* value
Age (y)	132	50 (27-83)	611	48 (24-83)	.02
FT3 (pg/mL)	132	7.2 (2.4-26.4)	611	6.5 (2.5-28.2)	.06
FT4 (ng/dL)	132	3.0 (1.1-7.8)	611	2.6 (0.9-7.8)	.03
TSH (mIU/L)	132	0.01 (0.01-2.82)	611	0.01 (0.01-3.93)	<.01
CRP (mg/dL)	115	4.6 (0.2-22.0)	526	3.1 (0.2-25.1)	<.01
TgAb (IU/mL)	132	14.0 (10-1015)	601	15.3 (10-2366)	.22
TgAb positivity, n (%)	132	28/132 (21.2%)	601	133/601 (22.1%)	.82
TPOAb (IU/mL)	132	9.9 (5-528)	601	10.5 (5-499.9)	.17
TPOAb positivity, n (%)	132	9/132 (6.8%)	601	36/601 (6.0%)	.69
Thyroid volume (mL)	76	27.0 (9.1-66.3)	345	23.4 (5.1-70.8)	<.01
PSL therapy, n (%)	132	100/132 (75.8%)	611	470/611 (76.9%)	.77
Interval from symptom onset to initiation of PSL (days)	89	21 (4-113)	418	18 (1-150)	<.05

Data are presented as median (range).

Abbreviations: CRP, C-reactive protein; FT3, free triiodothyronine; FT4, free thyroxine; PSL, prednisolone; TgAb, antithyroglobulin antibody; TPOAb, antithyroid peroxidase antibody; TSH, thyroid-stimulating hormone.

### Treatment of subacute thyroiditis

**Figure 2:**
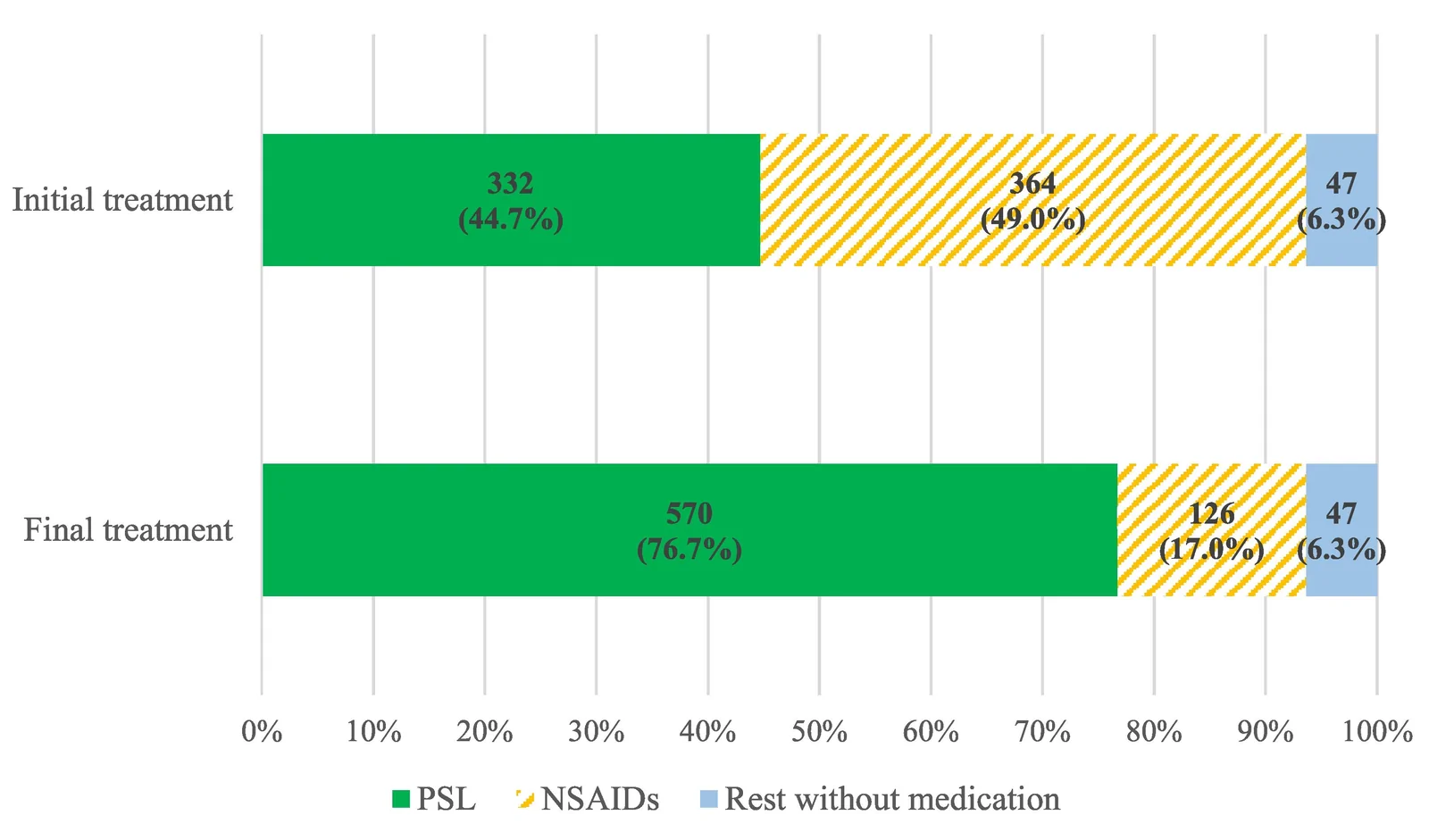
Initial and final treatment of SAT. NSAIDs, nonsteroidal anti-inflammatory drugs; PSL, prednisolone; SAT, subacute thyroiditis.

Initial treatment of SAT consisted of PSL therapy in 332 patients (44.7%), NSAIDs in 364 patients (49.0%), and rest without medication in 47 patients (6.3%). Of the 364 patients who initially received NSAIDs, 238 (65.4%) were later switched to PSL. As a result, the final treatment was PSL in 570 patients (76.7%), NSAIDs in 126 patients (17.0%), and rest only in 47 patients (6.3%) (Fig. 2).

The initial PSL dose was 15 (5-30) mg/day, the maximum PSL dose was 15 (5-30) mg/day, the duration of PSL therapy was 68 (2-417) days, and the cumulative PSL dose was 560 (30-3172) mg (Table 3). Initial PSL doses of 5, 10, 15, 20, 25, and 30 mg were administered to 3 (0.5%), 59 (10.4%), 378 (66.3%), 126 (22.1%), 2 (0.4%), and 2 (0.4%) patients, respectively. Of the 570 patients who received PSL therapy, 441 (77.4%) were treated within 90 days. In contrast, 9 patients were treated for 200 to less than 300 days, 1 patient for 300 to less than 400 days, and 1 patient for more than 400 days. All of these 11 patients were negative for both TgAb and TPOAb.

**Table 3:** PSL dose and treatment characteristics

	Total	E group	PH group	*P* value
Initial PSL dose (mg/day)	15 (5-30)	15 (5-30)	15 (10-30)	.76
Maximum PSL dose (mg/day)	15 (5-30)	15 (5-30)	15 (10-30)	.57
Duration of PSL therapy (days)	68 (2-417)	68 (2-417)	70 (31-210)	.46
Cumulative PSL dose (mg)	560 (30-3172)	560 (30-3172)	525 (329-1922.5)	.97
Interval from symptom onset to initiation of PSL (days)	n = 507	n = 493	n = 14	
	19 (1-150)	19 (1-150)	28 (8-63)	.09

Data are presented as median (range).

Abbreviations: E group, euthyroid group; PH group, permanent hypothyroidism group; PSL, prednisolone.

Adverse effects of PSL were observed in 7 of 570 patients (1.2%), including cystitis and perforated diverticulitis (n = 1), skin rash (n = 1), worsening of macular edema (n = 1), herpes zoster (n = 1), central serous chorioretinopathy (n = 1), and steroid-induced diabetes mellitus (n = 2).

Table 4 compares the baseline characteristics of patients who were treated with PSL as the final treatment and those who were not. Patients treated with PSL were significantly younger than those without PSL therapy (*P* < .01). In addition, they had significantly higher FT3 and FT4 levels, lower TSH levels, and higher CRP levels.

**Table 4:** Comparison of baseline characteristics between patients with and without PSL therapy

	PSL therapy	No PSL therapy	*P* value
Female/male, n	470/100	141/32	.77
Age (y)	47 (24-83)	51 (24-75)	<.01
FT3 (pg/mL)	6.9 (2.5-28.2)	5.4 (2.4-25.0)	<.01
FT4 (ng/dL)	2.7 (0.9-7.8)	2.3 (1.0-7.8)	<.01
TSH (mIU/L)	0.01 (0.01-3.93)	0.01 (0.01-2.82)	<.01
CRP (mg/dL)	3.5 (0.2-25.1)	1.8 (0.2-15.1)	<.01

Data are presented as median (range).

Abbreviations: CRP, C-reactive protein; FT3, free triiodothyronine; FT4, free thyroxine; PSL, prednisolone; TSH, thyroid-stimulating hormone.

### Thyroid function after recovery from subacute thyroiditis

After recovery from SAT, thyroid function was normal in 675 patients (90.8%), subclinical hypothyroidism with TSH < 10 mIU/L was observed in 36 patients (4.8%), and PH was seen in 32 patients (4.3%).

Of the 675 patients whose thyroid function normalized, 343 (50.8%) achieved normalization during SAT treatment. In those who normalized after the completion of treatment, the timing of normalization could be determined in 263 cases, and 239 (90.9%) of them achieved normal thyroid function within 9 months after treatment completion. Based on these results, PH in this study was defined as hypothyroidism that continued beyond 9 months after completion of treatment.

### Characteristics of permanent hypothyroidism in patients with subacute thyroiditis

**Figure 3:**
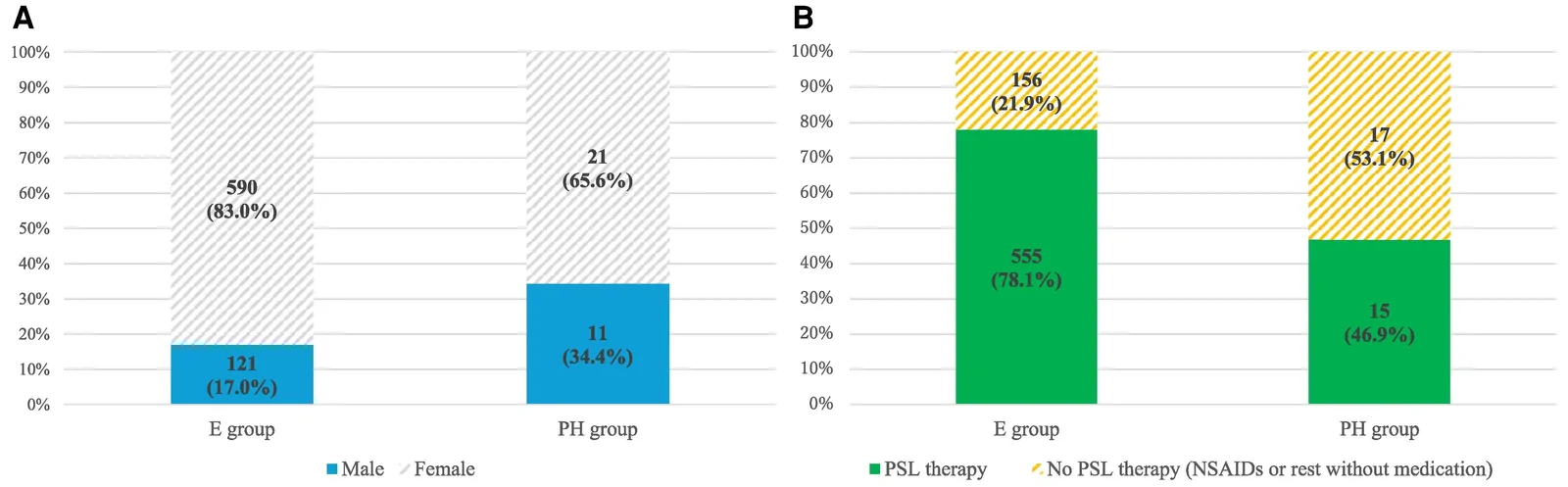
(A) Proportions of male and female patients in the E and PH groups. (B) Proportion of patients treated with PSL in the E and PH groups. E group, euthyroid group; NSAIDs, nonsteroidal anti-inflammatory drugs; PH group, permanent hypothyroidism group; PSL, prednisolone.

No significant differences were observed between the E and PH groups in terms of age, thyroid function (FT3, FT4, and TSH), CRP levels, TgAb and TPOAb levels and positivity rates, or thyroid volume (Table 1). The proportion of male patients was significantly higher in the PH group (11 of 32, 34.4%) than in the E group (121 of 711, 17.0%) (*P* = .01) (Fig. 3A).

### Relationship between prednisolone therapy and permanent hypothyroidism

The proportion of patients treated with PSL was significantly lower in the PH group (15 of 32, 46.9%) than in the E group (555 of 711, 78.1%) (*P* < .01) (Fig. 3B).

There were no significant differences between the E and PH groups in the initial PSL dose, the maximum PSL dose, the duration of PSL therapy, or the cumulative PSL dose (Table 3). In 507 of the 743 patients, the interval from symptom onset to initiation of PSL therapy could be determined. The median interval was 28 (8-63) days in the PH group and 19 (1-150) days in the E group; it tended to be longer in the PH group (*P* = .09).

### Risk factors for permanent hypothyroidism

Multivariable logistic regression analysis was performed to identify risk factors for PH, including PSL therapy, sex, and age. No PSL therapy (odds ratio [OR] 3.92, 95% confidence interval [CI] 1.88-8.24, *P* < .01) and male sex (OR 2.52, 95% CI 1.13-5.37, *P* = .03) were significantly associated with PH, whereas age was not (OR 0.99, 95% CI 0.96-1.02, *P* = .63) (Table 5). The primary findings were unchanged in the sensitivity analyses, with no PSL therapy and male sex remaining significantly associated with PH (Table 6).

**Table 5:** Multivariable logistic regression analysis of factors associated with PH

Variable	OR	95% CI	*P* value
No PSL therapy	3.92	1.88-8.24	<.01
Male sex	2.52	1.13-5.37	.03
Age	0.99	0.96-1.02	.63

Abbreviations: CI, confidence interval; OR, odds ratio; PH, permanent hypothyroidism; PSL, prednisolone.

**Table 6:** Sensitivity analyses of multivariable logistic regression models for PH

Model	No PSL therapy OR (95% CI)	Male sex OR (95% CI)	Age OR (95% CI)
Main model	3.92 (1.88-8.24)	2.52 (1.13-5.37)	0.99 (0.96-1.02)
Main model + FT4	4.10 (1.96-8.66)	2.38 (1.06-5.09)	0.99 (0.96-1.02)
Main model + FT4 + CRP	4.80 (2.15-10.89)	2.77 (1.15-6.32)	0.99 (0.96-1.03)
Patients aged <70 years	4.13 (1.83-9.34)	2.83 (1.15-6.59)	1.03 (0.99-1.08)

Abbreviations: CI, confidence interval; CRP, C-reactive protein; FT4, free thyroxine; OR, odds ratio; PH, permanent hypothyroidism; PSL, prednisolone.

### Comparison between patients aged <70 and ≥70 years

A comparison between patients aged <70 and ≥70 years is shown in Table 7. Of the 743 patients, 41 (5.5%) were aged ≥70 years. Among patients aged ≥70 years, FT3 and FT4 levels were significantly lower (*P* < .01 and *P* = .03, respectively), TSH levels were significantly higher (*P* = .02), and CRP levels were significantly lower (*P* = .02) than in patients aged <70 years.

**Table 7:** Comparison of clinical characteristics between patients aged <70 and ≥70 years

	ｎ	Age <70 years	Age ≥70 years	*P* value
Total, n	743	702	41	
Female/male, n	743	576/126	35/6	.68
FT3 (pg/mL)	743	6.7 (2.4-28.2)	5.4 (2.8-13.8)	<.01
FT4 (ng/dL)	743	2.7 (1.0-7.8)	2.3 (0.9-5.4)	.03
TSH (mIU/L)	743	0.01 (0.01-3.93)	0.02 (0.01-2.82)	.02
CRP (mg/dL)	641	3.3 (0.2-25.1)	1.6 (0.2-13.4)	.02
TgAb (IU/mL)	733	15.3 (10-2366)	11.8 (10-766)	.01
TgAb positivity, n (%)	733	153/693 (22.1%)	8/40 (20.0%)	.85
TPOAb (IU/mL)	733	10.4 (5.0-528)	10.0 (5.3-290)	.76
TPOAb positivity, n (%)	733	39/693 (5.6%)	6/40 (15.0%)	.03
PSL therapy, n (%)	743	547/702 (77.9%)	23/41 (56.1%)	<.01
Duration of PSL therapy (days)	570	69 (2-417)	57 (35-98)	.01
PSL therapy >90 days, n (%)	570	128/547 (23.4%)	1/23 (4.3%)	.04
Change from NSAIDs to PSL therapy, n (%)	364	232/345 (67.2%)	6/19 (31.6%)	<.01
Permanent hypothyroidism, n (%)	743	26/702 (3.7%)	6/41 (14.6%)	<.01

Data are presented as median (range).

Abbreviations: CRP, C-reactive protein; FT3, free triiodothyronine; FT4, free thyroxine; NSAIDs, nonsteroidal anti-inflammatory drugs; PSL, prednisolone; TgAb, antithyroglobulin antibody; TPOAb, antithyroid peroxidase antibody; TSH, thyroid-stimulating hormone.

Prednisolone therapy was used in 547 of 702 patients (77.9%) aged <70 years and in 23 of 41 patients (56.1%) aged ≥70 years (*P* < .01). The duration of PSL therapy was also significantly shorter in patients aged ≥70 years (*P* = .01). The proportion of patients receiving PSL for >90 days was significantly lower in those aged ≥70 years (1 of 23, 4.3%) than in those aged <70 years (128 of 547, 23.4%) (*P* = .04). In patients who initially received NSAIDs, the proportion who switched to PSL therapy was significantly lower in patients aged ≥70 years (6 of 19, 31.6%) than in those aged <70 years (232 of 345, 67.2%) (*P* < .01).

Permanent hypothyroidism was significantly more common in patients aged ≥70 years (6 of 41, 14.6%) than in those aged <70 years (26 of 702, 3.7%) (*P* < .01). In patients aged ≥70 years, PSL therapy was used in 2 of 6 patients (33.3%) in the PH group and in 21 of 35 patients (60.0%) in the E group (*P* = .38).

## Discussion

The main treatment for SAT is anti-inflammatory therapy. According to the 2016 ATA guidelines (11), NSAIDs are recommended for patients with mild symptoms, whereas glucocorticoids are advised for patients with moderate to severe symptoms or for those who do not respond to NSAIDs. The standard recommended dose is PSL 40 mg/day for 1 to 2 weeks, followed by a gradual taper over 2 to 4 weeks or longer, depending on the clinical response. Another recommended method is to start PSL at 15 mg/day and reduce the dose by 5 mg every 2 weeks (11, 12). In the present study, most patients started PSL at 15 mg/day (66.3%) or 20 mg/day (22.1%), and many cases followed the latter approach. In a previous report, about 80% of patients received PSL therapy within 8 weeks, and the longest duration was 40 weeks (12). In the present study, 77.4% of patients were treated within 90 days, and the longest duration was 417 days. The most important differential diagnosis of SAT is pHT. Similar to SAT, pHT is an inflammatory thyroid disease with a painful goiter, but it is usually more resistant to treatment and requires a longer treatment period (13). When the treatment period of SAT becomes prolonged, it can be difficult to distinguish from pHT. In the present study, 11 patients received PSL for more than 200 days, but all were negative for both TgAb and TPOAb, and therefore diagnosed with SAT.

Several studies have shown that glucocorticoid therapy relieves pain faster than NSAIDs (2, 14). In the present study, 65.4% of patients who started treatment with NSAIDs required switching to PSL because of insufficient efficacy, which was generally consistent with previous reports showing that approximately 60% of patients initially treated with NSAIDs subsequently required glucocorticoid therapy (5, 15).

Subacute thyroiditis often presents with thyrotoxicosis due to destructive thyroiditis at its onset. This is usually followed by a hypothyroid phase, and in most cases, thyroid function eventually returns to normal. However, some patients do not recover and develop PH. Several studies have reported the frequency of PH after recovery from SAT, ranging from 5% to 27% (11, 16). In the present study, the rate was 4.3%, slightly lower, but generally consistent with previous reports.

Some studies have suggested that the incidence of PH differs depending on the treatment used (2, 5, 7, 17). In the present study, no PSL therapy was identified as an independent risk factor for PH, suggesting that PSL therapy may reduce this risk. Yuan et al. reported a meta-analysis comparing the treatment outcomes of glucocorticoids and NSAIDs in patients with SAT and showed that glucocorticoid therapy was associated with a lower incidence of PH than NSAIDs, consistent with the present findings (17). In contrast, Fatourechi et al. reported that glucocorticoid therapy significantly increased the risk of PH (2), which was considered to reflect the greater severity of SAT in patients requiring such treatment. In the present study, despite higher FT3 and FT4 levels, lower TSH levels, and higher CRP levels indicating more severe disease, the rate of PH was lower in patients treated with PSL. These findings suggest that PSL therapy for SAT may have a thyroid-protective effect through its anti-inflammatory action and help prevent PH. In addition, the interval from symptom onset to initiation of PSL therapy was longer in patients with PH (*P* = .09). Early initiation of PSL therapy for SAT may help prevent PH. However, in the present study, the exact time of onset was unclear in many patients. Görges et al. reported that the length of time until diagnosis was not related to hypothyroidism in long-term follow-up (16).

When young patients develop PH, they require long-term treatment with LT4. In patients aged <70 years, even if treatment starts with NSAIDs, 67.2% need to switch to PSL due to insufficient effect, and many eventually require PSL therapy. Therefore, unless PSL is contraindicated or requires caution, early initiation may be preferable.

In the present study, male sex was identified as a risk factor for PH. Hypothyroidism, in general, is more common in female patients (18). Corsello et al. reported that male patients with SAT tend to have a higher risk of developing long-term hypothyroidism (4), which is consistent with the present findings. They suggested that estrogen's anti-inflammatory effects during viral infections might play a protective role in SAT (19). In the present study, compared with female patients, male patients had significantly higher FT4, lower TSH, and higher CRP levels, indicating stronger inflammation, which supports this idea. Although there was no difference between sexes in the use of PSL therapy, the interval from symptom onset to initiation of PSL therapy was longer in males. This delay may have led to more severe inflammation in males. In addition, male patients were significantly older than female patients, which might also be related.

In the present study, there were no significant differences between the PH and E groups in age, thyroid function, CRP levels, thyroid autoantibodies, or thyroid volume. Similarly, Saklamaz et al. reported no association between PH and inflammatory markers, thyroid function, or thyroid autoantibodies (20). In contrast, CRP levels, thyroid function parameters, TgAb, and TPOAb have also been reported as risk factors for PH (4-6).

Next, patients aged ≥70 years had lower FT3, FT4, and CRP levels and higher TSH levels than those aged <70 years, suggesting milder disease activity. Patients aged ≥70 years were less likely to receive PSL therapy and more often recovered with NSAID therapy alone. Nevertheless, the incidence of PH was higher in patients aged ≥70 years. This may be partly explained by the lower rate of PSL therapy in this age group. In addition, since TSH levels are generally higher in elderly persons (21, 22), their thyroid reserve capacity might have been lower.

Prednisolone therapy can sometimes be difficult in elderly patients because of comorbidities. As approximately half of patients aged ≥70 years recovered without PSL therapy, NSAID therapy may be appropriate for mild cases. However, even when PSL therapy was used in patients aged ≥70 years, difficult-to-manage cases were rare, and PSL therapy was discontinued within 90 days in 95.7% of patients. Therefore, PSL therapy may still be appropriate in severe cases when it is not limited by comorbidities or concerns about side effects.

This study has several limitations. First, it was a retrospective study. Treatment decisions were made individually at the discretion of the attending physicians. Second, many patients discontinued follow-up or were not monitored for thyroid function over a long period, leading to a high dropout rate. Third, some laboratory parameters had missing data. Fourth, many patients were not followed with ultrasound examinations after recovery from SAT, so ultrasound findings could not be analyzed. Fifth, because only 32 patients developed PH, the variables included in the multivariable logistic regression analysis were limited to sex, age, and PSL therapy to avoid overfitting. Although the primary findings were unchanged in the sensitivity analyses, the influence of other unmeasured factors, such as thyroid autoantibodies, treatment dose and duration, and thyroid volume, cannot be completely excluded.

## Conclusion

Prednisolone therapy for SAT has a thyroid-protective effect through its anti-inflammatory action and helps prevent PH. In younger patients, early initiation of PSL therapy may be preferable because PH can lead to long-term LT4 therapy and NSAIDs alone are often insufficient, except when PSL is contraindicated or requires caution.

In elderly patients, disease activity is milder, and about half recover without PSL; therefore, NSAID therapy is appropriate for mild cases. However, even when PSL therapy is used in elderly patients, few cases require prolonged treatment. Therefore, PSL therapy can be considered for severe cases when the risk associated with PSL therapy is low.

## Data Availability

Some or all datasets generated and/or analyzed during the current study are not publicly available but are available from the corresponding author on reasonable request.
